# Correlated iron isotopes and silicon contents in aubrite metals reveal structure of their asteroidal parent body

**DOI:** 10.1038/s41598-021-99160-9

**Published:** 2021-11-19

**Authors:** Soumya Ray, Laurence A. J. Garvie, Vinai K. Rai, Meenakshi Wadhwa

**Affiliations:** 1 School of Earth and Space Exploration, Arizona State University, Tempe, 85287 USA; 2 Center for Meteorite Studies, Arizona State University, Tempe, 85287 USA

**Keywords:** Planetary science, Geochemistry, Meteoritics

## Abstract

Iron isotopes record the physical parameters, such as temperature and redox conditions, during differentiation processes on rocky bodies. Here we report the results of a correlated investigation of iron isotope compositions and silicon contents of silicon-bearing metal grains from several aubritic meteorites. Based on their Fe isotopic and elemental Si compositions and thermal modelling, we show that these aubrite metals equilibrated with silicates at temperatures ranging from ~ 1430 to ~ 1640 K and likely sampled different depths within their asteroidal parent body. The highest temperature in this range corresponds to their equilibration at a minimum depth of up to ~ 35 km from the surface of the aubrite parent body, followed by brecciation and excavation by impacts within the first ~ 4 Myr of Solar System history.

## Introduction

Metal segregation in accreting rocky bodies occurred very early on in the history of the Solar System, in some cases within 1 Myr after the formation of calcium-aluminum-rich inclusions (CAIs) which are the oldest known solids to have formed in the solar nebula and are, therefore, thought to represent the time of Solar System formation^1–3^. While magmatic iron meteorites sample cores of differentiated asteroidal bodies and provide insights into the processes and timescales of core formation^3–5^, achondritic meteorites are key for providing complementary understanding of silicate differentiation and crust formation on such bodies^6^. In particular, the stable isotopic compositions of differentiated meteorites and their components have added to our understanding of the physical parameters, such as temperature, pressure, and redox conditions relevant to differentiation processes on planetesimals in the early Solar System^7, 8^.

Iron is the most abundant multi-valent element in planetary reservoirs. Its abundance and variable oxidation states (i.e., Fe^0^ in metal, Fe^2+^ in silicates/sulfides, and Fe^3+^ in silicates/oxides) have allowed it to be used in studies aimed at understanding the effects of metal-silicate equilibration on stable Fe isotope compositions^9–17^. During equilibration of metal with silicates, the Fe isotope composition (expressed as δ^56^Fe, which is the deviation in parts per thousand of the ^56^Fe/^54^Fe ratio of the sample relative to the IRMM-014 bracketing standard) of metal is expected to be enriched in the heavier isotopes of Fe compared to coexisting silicates. This expectation stems from crystal chemical principles involving an interplay of bond strength, bond length, bond stiffness, coordination number, valence, and associated vibrational frequencies that govern the occupation of crystallographic sites by heavy or light isotopes^18, 19^. A short and stiff bond is expected to accommodate the heavier isotopes of a given element. The presence of delocalized electrons in Fe-metal facilitates shorter and stiffer metallic bonds, compared to the Fe–O bonds in silicates, which favors the enrichment of heavier Fe isotopes in the Fe-metal^19^. This is consistent with the observation that metals from pallasites^9^, enstatite chondrites^13^, aubrites^13, 17^, iron meteorites^20^, and ordinary chondrites^21–23^ are characterized by heavier Fe isotope compositions compared to coexisting silicates and sulfides. This further leads to the expectation, supported by some recent experimental studies^14–16^, that the metallic core of a differentiated body should have a heavier Fe isotope composition compared to its silicate mantle.

However, there is no detectable fractionation of Fe isotopes between coexisting metal and silicate in the run products from at least some experiments^11, 12^. Recently*,* the effect of Si content of metal on Δ^56^Fe_metal-silicate_ (i.e., δ^56^Fe_metal_–δ^56^Fe_silicate_) was studied^16^. This study suggested that a combined substitution of Ni, S, and Si in the Fe-metal would lead to a heavier Fe isotope composition of the metal compared to the silicate. In contrast, based on nuclear resonant inelastic X-Ray scattering (NRIXS) measurements of mean force constants of Fe bonds in basaltic glass and metal alloys (such as Fe–Ni–Si, Fe–Si, and Fe–S), no significant fractionation between silicate and metal at pressures of up to 206 GPa was found^24^. Thus far, to our knowledge, there have been no studies that have investigated the potential correlation of Si content with Fe isotope fractionation in natural metallic samples that record metal-silicate equilibration.

Aubrites are pyroxenitic achondrites that are known to contain Si-bearing Fe–Ni metal. The pattern of siderophile element abundances in these Si-bearing aubrite metals reflects their residual nature, i.e., they represent metal that was trapped in the silicate-sulfide magma during partial melting and inefficient metal segregation (i.e., core formation) on the aubrite parent body^25^. Therefore, aubrites provide the opportunity to study the correlation between Si content and δ^56^Fe of these metals that are thought to have equilibrated with the surrounding silicates. Aubrites are dominated by almost FeO-free enstatite, with lesser amounts of diopside, forsterite, troilite, Si-bearing Fe–Ni metal, and a variety of accessory minerals that reflect the highly reducing conditions during their formation^26^. Aubrites are thought to represent the differentiated products of enstatite chondrite-like starting materials^27–29^ and are postulated to have originated on at least two distinct parent bodies: the main aubrite parent body, from which most aubrites are thought to have originated, and the Shallowater parent body^26, 30^. Most aubrites from the main aubrite body are fragmental or regolith breccias, while Shallowater is unbrecciated^26^. The unique unbrecciated, metal-rich Mount Egerton aubrite has been considered to either represent the core-mantle boundary of the main aubrite parent body^31^ or have originated on a distinct parent body^32^.

The Si content of Fe-metal is strongly temperature-dependent^33, 34^, and has been used to determine the equilibration temperatures in enstatite meteorites^13, 17, 35, 36^. However, these studies used a single Si content for all metals within a given aubrite to derive a metal-silicate equilibration temperature for that sample^13, 17, 36^. For example, equilibration temperatures were calculated to be ~ 1060 K for 2 mol% Si in metal for Shallowater^13^, ~ 1200 K^36^ for 4.5 mol% Si in metal or ~ 1460 K^17^ for 2 wt% Si in metal for Mount Egerton, and ~ 1130 K^36^ for 2.1 mol% Si in metal or ~ 1415 K^17^ for 0.91 wt% Si in metal for Norton County. However, the Si contents of different metal grains within a given aubrite have been shown to vary significantly^25, 37^. This variability may indicate that individual metal grains equilibrated with silicates over a range of temperatures and/or redox conditions which may additionally be reflected in their Fe isotope compositions. Here, we address the question of the origin of metal grains in the aubrites based on their major element composition, with a focus on Si, coupled with their δ^56^Fe. Since the elemental composition of metal, especially Si content, and δ^56^Fe are likely to be functions of temperature and redox conditions during metal-silicate equilibration, a correlation, if present, would provide insights into the physical conditions governing the formation of aubrites. Furthermore, we have modeled the thermal evolution of the aubrite parent body which allows us to infer the physical setting of the metal grains in the aubrites studied here and has implications for the structure of their parent body.

## Results

### Elemental and iron isotope compositions of aubrite metals

**Figure 1:**
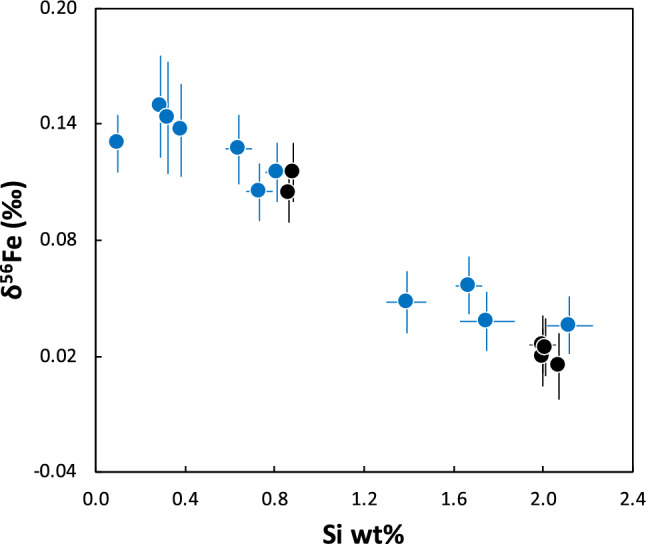
δ^56^Fe versus Si content of aubrite metals. Blue symbols represent metals from brecciated aubrites (Norton County and Bishopville) and black symbols show metals from non-brecciated aubrites (Shallowater and Mount Egerton). An inverse correlation between the δ^56^Fe and Si content of aubrite metals is observed. All errors are 2SE.

We measured the elemental abundances (Fe, Ni, Co, P, and Si) and Fe isotope compositions of ten metal grains from Norton County, four from Mount Egerton, one from Bishopville, and two from Shallowater. These aubrite metals have Si contents ranging between 0.1 and 2.12 wt% and δ^56^Fe values ranging from 0.015 ± 0.018 to 0.149 ± 0.026‰ (Table 1). Moreover, the δ^56^Fe values of these metal grains are inversely correlated with their Si contents (Fig. 1). There appears to be no strong correlation between the abundances of other elements (Fe, Ni, Co, P) and δ^56^Fe for the metal grains studied here (Supplementary Fig. S3). There are two previous studies^13, 17^ that report the Fe isotope compositions of aubrite metals. Unlike our study, neither of these studies^13, 17^ analyzed the Si content in the aubrite metal. In one of these studies^13^, two magnetic fractions (presumably enriched in metal) separated from Shallowater have δ^56^Fe of 0.039 ± 0.044‰ and 0.075 ± 0.059‰, and Fe contents of 46.4 wt% and 30.1 wt%, respectively. These magnetic fractions have lower δ^56^Fe values and Fe contents than the Shallowater metal grains analyzed here (Table 1), suggesting that phase(s) with lower δ^56^Fe and Fe content than metallic Fe–Ni contributed to these magnetic fractions^13^. Four metal samples from Norton County with δ^56^Fe_avg._ = 0.025 ± 0.012‰ (2SE) and two metal samples from Mount Egerton with δ^56^Fe_avg._ = 0.002 ± 0.017‰ (2SE) were reported in the other previous study^17^. These Fe isotope compositions are similar to some of those from Norton County (NC8–10) and Mount Egerton (ME1–4) reported here, but distinct from those of other aubrite metals reported in our study (Table 1).

**Table 1:** Elemental and Fe isotope compositions of the metal grains from the Norton County (NC), Mount Egerton (ME), Bishopville (BSP), and Shallowater (SW) aubrites.

Samples	Size* (mm)	Fe	Ni	Co	P	Si	δ^56^Fe ± 2SE	δ^57^Fe ± 2SE	n	$\Delta$ ^56^Fe ± 2SE	$\Delta$ ^57^Fe ± 2SE	T(K)	*f*O_2_ (ΔIW)
NC2 (100)	2	92.14	7.14	0.42	0.04	0.81	0.115 ± 0.010	0.157 ± 0.027	6	0.151 ± 0.018	0.215 ± 0.033	1559 ± 28	− 5.5
NC3 (100)	2	93.26	6.02	0.47	0.07	0.38	0.137 ± 0.024	0.197 ± 0.039	6	0.173 ± 0.020	0.255 ± 0.043	1510 ± 27	− 5.5
NC4 (100)	3	94.30	5.64	0.36	0.10	0.29	0.149 ± 0.026	0.292 ± 0.024	12	0.185 ± 0.030	0.350 ± 0.031	1493 ± 26	− 5.5
NC6 (100)	3	94.42	5.85	0.38	0.09	0.32	0.143 ± 0.029	0.222 ± 0.035	6	0.179 ± 0.033	0.280 ± 0.040	1499 ± 26	− 5.5
NC7 (200)	8	91.93	5.81	0.32	0.10	1.67	0.057 ± 0.012	0.082 ± 0.023	12	0.093 ± 0.019	0.140 ± 0.024	1608 ± 30	− 5.5
NC8 (200)	21	91.22	6.51	0.28	0.08	1.39	0.048 ± 0.016	0.066 ± 0.018	12	0.084 ± 0.022	0.124 ± 0.028	1596 ± 30	− 5.5
NC9 (200)	14	91.38	6.33	0.33	0.06	2.12	0.036 ± 0.015	0.068 ± 0.027	13	0.072 ± 0.021	0.126 ± 0.033	1626 ± 31	− 5.5
NC10 (200)	7	90.56	6.67	0.35	0.05	1.75	0.038 ± 0.014	0.086 ± 0.023	12	0.074 ± 0.021	0.144 ± 0.028	1613 ± 30	− 5.5
NC11 (200)	15	91.16	7.11	0.32	0.09	0.64	0.127 ± 0.018	0.248 ± 0.062	12	0.163 ± 0.023	0.306 ± 0.065	1545 ± 28	− 5.5
NC12 (200)	18	89.91	8.07	0.30	0.09	0.73	0.105 ± 0.014	0.218 ± 0.037	12	0.141 ± 0.021	0.276 ± 0.042	1555 ± 28	− 5.5
ME1 (200)	15	91.55	5.52	0.36	0.07	2.00	0.026 ± 0.010	0.067 ± 0.025	6	0.064 ± 0.012	0.119 ± 0.028	1633 ± 31	− 5.4
ME2 (200)	11	90.71	6.94	0.34	0.07	2.00	0.020 ± 0.014	0.061 ± 0.030	6	0.058 ± 0.016	0.113 ± 0.032	1634 ± 31	− 5.4
ME3 (200)	12	91.07	6.69	0.34	0.08	2.01	0.025 ± 0.015	0.057 ± 0.023	6	0.063 ± 0.017	0.109 ± 0.020	1633 ± 31	− 5.4
ME4 (200)	10	91.51	6.15	0.34	0.09	2.07	0.015 ± 0.018	0.062 ± 0.028	6	0.053 ± 0.019	0.114 ± 0.030	1635 ± 31	− 5.4
BSP3 (40)	1	93.44	6.03	0.35	0.06	0.10	0.130 ± 0.015	0.170 ± 0.036	6	–	–	1432 ± 24	− 5.5
SW1 (40)	1	93.31	5.24	0.37	0.36	0.89	0.115 ± 0.010	0.154 ± 0.023	6	0.094 ± 0.060	0.225 ± 0.109	1541 ± 28	− 5.7
SW3 (40)	1	88.98	8.61	0.30	0.24	0.86	0.104 ± 0.010	0.137 ± 0.023	6	0.083 ± 0.060	0.137 ± 0.109	1544 ± 28	− 5.7

Elemental concentrations are in wt% and are obtained by electron probe microanalysis (EPMA). Numbers within parentheses indicate number of EPMA data points averaged to obtain the elemental abundances of Fe, Ni, Co, P, and Si for each nodule. The $\Delta$^56^Fe _metal-silicate_ values are calculated using the δ^56^Fe_metal_ from this study and published values^13, 17^ of δ^56^Fe_silicate_ for that particular meteorite; $\Delta$^57^Fe_metal-silicate_ is calculated similarly (note that δ^56^Fe_silicate_ and δ^57^Fe_silicate_ for BSP do not exist, so $\Delta$^56^Fe and $\Delta$^57^Fe for this aubrite are not calculated). Calculated equilibration temperature (T in K) and *f*O_2_ (ΔIW) for each of these aubrite metals are also shown.

*Size refers to the longest axis (in most cases, diameter) of the metal grain in mm.

### Equilibration temperatures and oxygen fugacities

**Figure 2:**
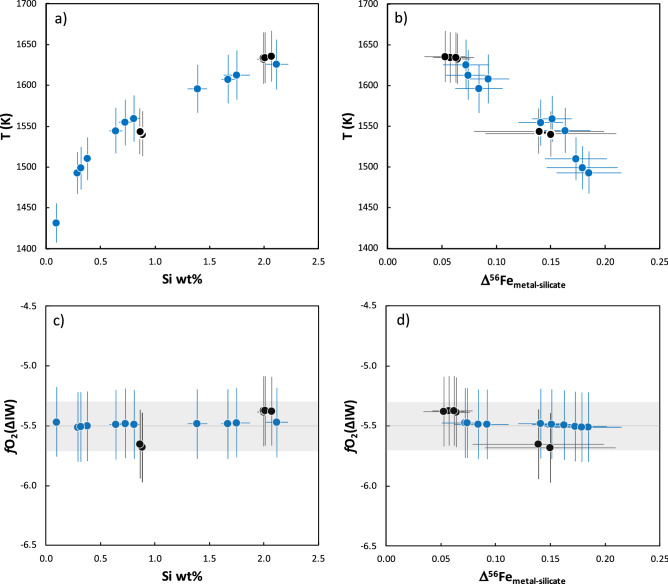
Plots of (**a**) calculated metal-silicate equilibration temperature (T in Kelvin) vs. measured Si content (wt%); (**b**) calculated metal-silicate equilibration temperature (T in Kelvin) vs. Δ^56^Fe_metal-silicate_; (**c**) calculated *f*O_2_ $\left( {{{\Delta \text IW}}} \right)$ vs. Si content (wt%); and (**d**) calculated *f*O_2_ $\left( {{{\Delta \text IW}}} \right)$ vs. Δ^56^Fe_metal-silicate_ for aubrite metals. The horizontal grey line and box in 2c and 2d represent the average ± 2SD (i.e.,–5.5 ± 0.2) of the calculated *f*O_2_ for the aubrite metals. The Δ^56^Fe_metal-silicate_ (δ^56^Fe_metal_–δ^56^Fe_silicate_) values are calculated using δ^56^Fe_metal_ from this study and δ^56^Fe_silicate_ for Norton County, Mount Egerton, and Shallowater from previous studies^13, 17^; the Bishopville data point is not shown in 2b and 2d because the δ^56^Fe_silicate_ value for this sample has not been reported and the Δ^56^Fe_metal-silicate_ value could not be calculated. The symbols are same as in Fig. 1.

Based on the elemental compositions of the metal grains and silicates from each of the aubrites studied here, we calculated the metal-silicate equilibration temperatures and *f*O_2_ values (see “Methods” section below for the equations and parameters used for these calculations). The calculated equilibration temperatures (Table 1, Fig. 2a,b) based on partitioning of Si and Fe between metal and silicate range from ~ 1432 to ~ 1635 K. The four metal grains from Mount Egerton (ME1–4) record the highest equilibration temperatures that are the same within the errors (Table 1). The metals from Shallowater (SW1 and SW3) record lower equilibration temperatures that are also, within the errors, similar to each other (Table 1). However, the 10 metal grains from Norton County record a temperature range of about ~ 130 K, from 1493 K in NC4 to 1626 K in NC9. Calculated *f*O_2_ values (Table 1, Fig. 2c,d) are similar within the errors, with an average value of –5.5 ± 0.2 (2SD) log units relative to the iron-wüstite buffer (IW); these values are similar to those previously reported for aubrites^38^.

### Modeling the thermal evolution of the aubrite parent body

**Figure 3:**
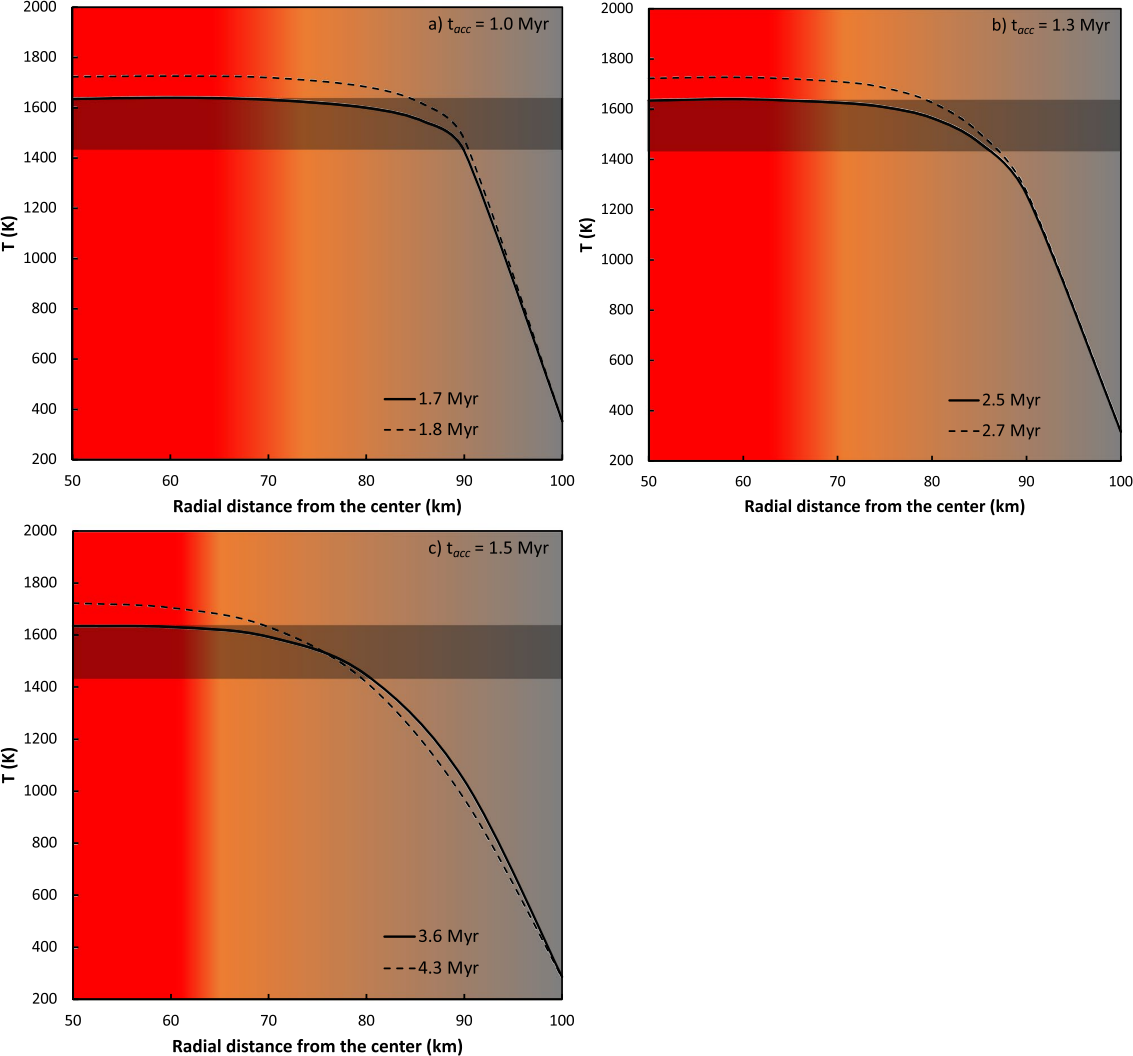
The calculated temperature (T in Kelvin) versus radial distance (r in km) from the center of the 200 km diameter aubrite parent body (AuPB) at 1 AU from the Sun at different times (shown by solid and dashed curves) assuming that the time of accretion was (**a**) 1.0 Myr; (**b**) 1.3 Myr; and (**c**) 1.5 Myr after CAI formation. The parameters used for these calculations are listed in Supplementary Table S3. Warmer colors represent higher temperatures with increasing depth from the surface of the AuPB. The black solid curve corresponds to the thermal profile with interior T of 1635 K (i.e., the highest estimated metal-silicate equilibration temperature recorded in the aubrite metals in this study). The black dashed curve represents the thermal gradient with interior T of 1723 K beyond which > 50% silicate melting would lead to obliteration of the thermal gradient in the AuPB (see text for details). Horizontal gray bar illustrates the range of metal-silicate equilibration temperatures (1432–1635 K) estimated for the aubrite metals in this study. The thermal gradient within the AuPB inferred from the metal-silicate equilibration temperatures recorded by the aubrite metals in this study is attained by (**a**) 1.7–1.8 Myr (for t_acc_ = 1.0 Myr), (**b**) 2.5–2.7 Myr (for t_acc_ = 1.3 Myr), and (**c**) 3.6–4.3 Myr (for t_acc_ = 1.5 Myr) after CAI formation**.** Therefore, the aubrite parent body likely acquired this thermal gradient well within 4.3 Myr after CAI formation (assuming its accretion occurred within ~ 1.5 Myr after CAI formation^42^). Note that the required minimum depth of excavation increases with increasing accretion time, with an excavation depth of at least ~ 35 km for an accretion time of ~ 1.5 Myr after CAI formation.

Heating and melting processes on early-formed planetesimals are thought to be driven largely by the decay of the short-lived radionuclide ^26^Al^39^. The evolution of the interior temperatures of parent bodies can be calculated using the analytical solution to the partial differential equation for heat conduction based on estimates of the ^26^Al content at the time of accretion assuming a constant heat capacity^40, 41^ (see “Methods” section below for equations and parameters used for these calculations). Since we are only interested in a first-order relationship between the accretion timescales and evolution of interior temperatures, we have assumed constant heat capacity (*C*_*P*_), thermal conductivity (K), density ($\rho$), and other appropriate parameters for the aubrite parent body (Supplementary Table S3) to calculate temperatures (T in Kelvin) as a function of distance from the center (r in km) of the parent body at different times. For these calculations, we assume that the latest accretion time for the aubrite parent body was ~ 1.5 Myr after CAI formation^42^; as such, for illustrative purposes, we have performed the calculations described above for accretion times of 1.0, 1.3, and 1.5 Myr after CAI formation. Figure 3 shows that a temperature profile relevant to the range of metal-silicate equilibration temperatures recorded by the aubrite metals studied here can be attained within 4.3 Myr after CAI formation. Moreover, the highest recorded equilibration temperature is achieved at a minimum depth of up to ~ 35 km from the surface of the parent body (at the latest estimated accretion time of ~ 1.5 Myr after CAI formation).

## Discussion

The degree of mass-dependent fractionation of Fe isotopes between metal and silicates is expected to decrease with increasing temperature of equilibration^18, 19, 43, 44^. In addition, metal-silicate equilibration experiments demonstrate increased partitioning of Si in metal with temperature^33, 34^. We hypothesize that the anti-correlation between Si content and δ^56^Fe of aubrite metals (Fig. 1) can be explained in terms of their dependence on the metal-silicate equilibration temperatures. The equilibration between metal and silicates in aubrites can be expressed by the following reaction:

1 $${\text{SiO}}_{2} + 2{\text{Fe}}_{{{\text{metal}}}} = 2{\text{FeO}} + {\text{Si}}_{{{\text{metal}}}}$$

Previous estimates^13, 36^ of metal-silicate equilibration temperatures for aubrites were considered to be too low^17^ given the temperatures required for the onset of melting for an enstatite chondrite composition^45, 46^. Here, we calculate the metal-silicate equilibration temperatures based on the solubility of Si in Fe-metal (Table 1; Fig. 2a,b) using an equation utilized previously^34^.

Prior studies have discussed the control of *f*O_2_ on mass-dependent fractionation of Fe isotopes of various planetary materials^47–49^. In general, an ion with a higher valence is preferentially enriched in the heavier isotopes of that element^18, 19^. A linear positive correlation between the Fe force constant and $\sum {\text{Fe}}^{3 + } /{\text{Fe}}_{total}$ in synthetic silicate was observed^49^. Force constants are measures of bond strengths and a higher force constant translates to stronger bonds, which in turn facilitates the enrichment of heavier isotopes. Therefore, a higher force constant for a higher $\sum {\text{Fe}}^{3 + } /{\text{Fe}}_{total}$ predicts that Fe^3+^-bearing phases are more enriched in the heavier Fe isotopes compared to Fe^2+^-bearing phases. It was, however, suggested that only 1/3 of the Fe isotope fractionation between mid-oceanic ridge basalts and terrestrial mantle could be explained due to the change in oxidation state of Fe in basaltic melt, while a change in the structural environment of the silicate melt was invoked to explain the remaining offset^49^. Given their highly reduced nature (and the resulting absence of Fe^3+^) of aubrites, the increasingly lighter δ^56^Fe values of metals correspond to smaller Δ^56^Fe_metal-silicate_ values that are likely a function of the change in Fe-bonding environment with increasing temperature and Si content. For ordinary chondrites (OCs), the δ^56^Fe of metal increases from H (most reduced OCs) through L to LL (most oxidized OCs), although a temperature dependence related to petrographic grade is also observed^21, 22^. Studies have also shown the increased partitioning of Si in metal with decreasing oxygen fugacity^34, 45, 46^. To assess the effect of redox on the Fe isotope fractionation recorded in the aubrite metals, we also calculated *f*O_2_ values for aubrites. The aubrite metals show a narrow range of *f*O_2_ from –5.4 to –5.7 log units relative to the iron-wüstite buffer (${\text{IW}}$) (Table 1); these values are similar to those previously reported for aubrites^38,^ suggesting that the primary control on the Si content and the Fe isotopic composition of metal in the aubrites is the metal-silicate equilibration temperature. We conclude that the observed anti-correlation between Si content and δ^56^Fe in the Si-bearing metals from the aubrites studied here (Fig. 1) is consistent with these metals recording a range of metal-silicate equilibration temperatures (Fig. 2a,b), with little variation in the redox conditions (Fig. 2c,d).

The incorporation of Si along with other elements such as Ni and S in metal has the potential to alter the bonding environment of Fe, such as bond strength, length, and stiffness. However, a series of piston cylinder experiments at 1850 °C and 1 GPa were unable to detect any resolvable metal-silicate Fe isotope fractionation with varying Si content in the metal, even though a change in bond length with incorporation of Si in metal was invoked^16^. The radial distribution function (RDF) or probability of finding another atom in a spherical shell of radius *r* in iron alloys with 17 wt% Si between 0–3 GPa and 1570–2040 K reveals that the packing structure around Fe does not change significantly from that of pure Fe-liquids^50^. Although the first neighbor interatomic distance (*r*_*1*_) decreases slightly from 2.56–2.57 Å in pure Fe melt to 2.54–2.53 Å in the alloy and is invariant with respect to pressure (P) or temperature (T), large structural changes in Fe–Si alloys compared to pure Fe-metal are not predicted^50^. Therefore, it seems unlikely that greater incorporation of Si in aubrite metal is the cause of the progressively lighter δ^56^Fe of such metal, especially since the Si content of aubrite metal is much lower ($\le$ 2.12 wt% Si) compared to that in the studied Fe–Si alloy (17 wt% Si)^50^.

A linear relationship between force constants of Fe bonds and pressure based on the NRIXS technique  is reported for Fe-alloys^24^. Force constants, which are a measure of bond strength, can be used to derive reduced partition function ratios, also called *β*-factors, which in turn can be used to calculate the Fe isotope composition at a given temperature. Based on the linear relationship between force constant ($\langle F\rangle$) and pressure (P), i.e.,

2 $$\langle F\rangle = a*{\text{P}} + b$$

and the constants *a* and *b* corresponding to Fe-alloys^24^, we find that the values for $\langle F\rangle$ of Fe bonds in Fe_86.8_Ni_8.6_Si_4.6_ (174.20 ± 6.50 N/m) and Fe_92_Ni_8_ (167.00 ± 17.30 N/m) are similar at a pressure of ~ 0 GPa (appropriate for the interior of the aubrite parent body). As such, no resolvable difference is expected in the *β*-factors of Fe bonds in Fe_86.8_Ni_8.6_Si_4.6_ and Fe_92_Ni_8_ alloys. This suggests that at the same temperature, fractionation of Fe isotopes between Fe–Ni–Si or Fe–Ni alloy and silicate will be indistinguishable under the low-pressure conditions relevant to asteroid-sized bodies. We propose here that the range of equilibration temperatures recorded by the aubrite metals (Table 1; Fig. 2a,b) is likely related to metal-silicate equilibration at different depths within the aubrite parent body in the early Solar System. This could be the result of inefficient separation of metal during partial melting and differentiation on the aubrite parent body^25^. As such, residual metal at greater depths experienced a higher equilibration temperature and therefore would have a higher Si content and smaller Fe isotope fractionation (relative to silicates) compared with metals that equilibrated at shallower depths.

Several experiments have demonstrated the importance of oxygen concentration in lowering the dihedral angle in Fe–O–S liquids, formation of an interconnected metallic melt network, and percolation of metal in relatively oxidized bodies^51–53^. However, under reducing conditions such as those that existed on the aubrite parent body(s), the presence of Si in metal does not reduce its surface tension which consequently increases the dihedral angle above the 60° threshold^54^. This high angle prevents the formation of an interconnected metallic melt network until the percolation threshold is exceeded^55, 56^. Once this percolation threshold is exceeded, the metal segregates. But eventually, the melt network is again disconnected as the melt fraction decreases below this threshold; e.g., ~ 1–2% metal remains trapped in the silicate melt^56^. These trapped pockets of residual metal subsequently equilibrate with the surrounding silicates. The formation of a metallic core in the aubrite parent body^13, 25, 57^ likely occurred through silicate melting and not percolation alone^54^. Melting experiments conducted on the Indarch EH4 chondrite show onset of melting at 1273 K and complete melting of silicates at 1773 K at ambient pressure^45^; complete melting of such a composition at 1 GPa occurs at 1873 K^46^. Based on these melting experiments^45, 46^, the calculated equilibration temperatures of between 1432 and 1635 K for the aubrite metals in this study suggest ~ 7–20% partial silicate melting in the aubrite parent body. This degree of silicate partial melting is insufficient compared to the ~ 50% silicate melting required for complete segregation of metal^55^ (possibly leading to the inefficient separation of metal). At 1723 K, ~ 50% silicate melting occurs^45^ which leads to loss of shear strength due to convective motion^40^ and efficient segregation of metal^55^. Therefore, we consider 1723 K as an upper limit for our calculations of the thermal profile within the aubrite parent body (Fig. 3) as a further increase in temperature would lead to the near-complete melting of the aubrite parent body^45^, onset of convective motion obliterating any thermal gradient^40^, and efficient separation of metal^55^. Our calculations indicate that the thermal profile relevant to the equilibration temperature range recorded by the Si-bearing aubrite metals can be attained within 4.3 Myr after CAI formation, at a minimum depth of up to ~ 35 km from the surface (Fig. 3). This time interval is consistent with the ^26^Al–^26^Mg age of ~ 2.9 Myr after CAI formation^58^, the ^129^I–^129^Xe age of 4.6 ± 0.3 Myr after CAI formation for the crystallization of aubrite pyroxene^59^, and the ^53^Mn–^53^Cr age of ~ 4 Myr after CAI formation for the last Mn/Cr fractionation (i.e., global differentiation) event on the aubrite parent body^60^. Subsequent large-scale impacts while the parent body was still hot would have brought the metal grains from a range of depths to the surface, where they would have undergone rapid cooling and retained the Si contents and Fe isotope compositions they acquired during metal-silicate equilibration. Indeed, previous studies have suggested that the aubrite parent body(s) underwent a globally disruptive collision and subsequent reassembly to form a “rubble pile” asteroid^27, 30^.

**Figure 4:**
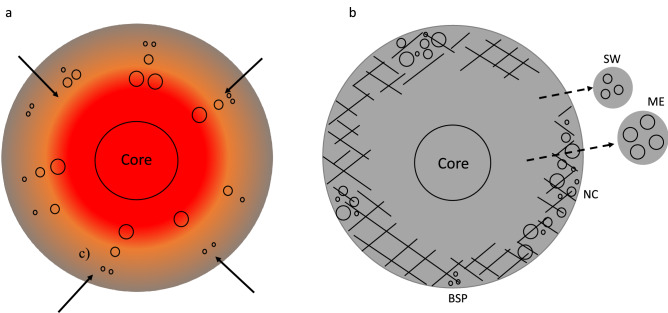
(**a**) Schematic illustration of the aubrite parent body (AuPB) at a time within 4.3 Myr after CAI formation, with warmer colors indicating higher temperatures. Silicon contents and Fe isotope compositions of metal grains in aubrites likely record a thermal gradient with depth from surface (larger circles represent metal grains recording higher equilibration temperatures). Impacts (shown as arrows) at this time, while the AuPB was still hot, could have excavated metals from depths of up to ~ 35 km from the surface. As a result, these excavated metals would have cooled quickly, quenching and preserving the Si contents and δ^56^Fe values at their respective metal-silicate equilibration temperatures. (**b**) Schematic illustration of the AuPB following impacts that excavated and quenched metals from different depths (and thus record different equilibration temperatures); such metals occur together in the brecciated aubrites, such as Norton County (NC) and Bishopville (BSP), that likely formed at the surface of the main AuPB. The non-brecciated aubrites Mount Egerton (ME) and Shallowater (SW) contain metals that only record equilibration at a particular depth (and equilibration temperature) at their sampling scale. It is possible that these samples may have been ejected altogether from the main AuPB by impacts (and thus did not form as part of near-surface breccias) or originated on distinct parent bodies that were disrupted by impacts.

In such a scenario, the brecciated aubrites would be expected to contain metals that equilibrated at different depths and were mixed together on the size-scale of the known meteorite sample. This is thought to be the case for the Norton County aubrite. This 1-ton meteorite provides abundant material for study, and over half of the metal grains studied here are from this sample. In contrast, the known masses of the unbrecciated aubrites Shallowater and Mount Egerton would be expected to only sample material from specific (shallower and deeper, respectively) depths of their parent body(s) (Fig. 4). Lack of brecciation (and thus no mixing of populations from different depths) explains the uniformity of the Si contents and δ^56^Fe values in different metal grains in each of these samples.

Shallowater^30^ and Mount Egerton^32^ have been proposed to belong to parent bodies distinct from the main aubrite body (from which Norton County and Bishopville originated). In fact, Shallowater is suggested to have undergone a complex cooling history, the first stage of which involved fast cooling from well above ~ 1273 K, most likely due to a low-velocity collision, breakup, and reassembly of a partly molten parent body^30^. Irrespective of whether or not these unbrecciated aubrites originated on the main aubrite parent body, the anti-correlation between the Si content and δ^56^Fe holds true for all the aubrite metals studied here and likely implies broadly similar cooling and disruption histories. Furthermore, given that our study shows that brecciated aubrite metals record equilibration with silicates over a range of temperatures, one cannot assume a single equilibration temperature to estimate the metal-silicate Fe isotope fractionation factor from such samples.

## Methods

### Bulk elemental composition and iron isotope measurements

Bulk compositions were measured by wavelength-dispersive spectroscopy (WDS) using a JEOL JXA-8530F electron microprobe in the Eyring Materials Center at Arizona State University (ASU) and a CAMECA SX100 electron microprobe in the Michael J. Drake Electron Microprobe laboratory at University of Arizona. Standards used for calibration are Fe metal, schreibersite (P), Ni metal, Co metal, troilite (S), and elemental Si. Bulk elemental abundances (Table 1) were acquired using a defocused 100 μm diameter beam at operating conditions of 20 kV and 20 nA. Elemental abundances for each metal are reported as averages of 40 to 200 analyzed points along transects across the metal. Accuracy and precision of our analyses were verified by comparing the elemental compositions of the Horse Creek, Monahans, and Santiago Papasquiero iron meteorites with published values. The samples studied here include 17 metal grains from four aubrites: ten from Norton County (NC2–NC4, NC6–NC12), four from Mount Egerton (ME1–4), two from Shallowater (SW1, SW3), and one from Bishopville (BSP3). Each metal grain was cut in half using a diamond-plated wafering blade; one half of each metal was used to create a polished mount for electron microprobe study (Supplementary Fig. S1).

The half of each small metal grain not embedded in epoxy was abraded using sandpaper and inspected under the microscope to ensure it was free of adhering silicates and rust. For the largest metal grains (NC7–12; ME1–4), a clean interior piece was cut using the diamond-plated wafering blade. The cleaned metal pieces were ultrasonicated in methanol, dried, and then dissolved in concentrated HCl. After careful inspection to ensure complete dissolution, solutions were then purified via column chromatography to separate Fe for isotopic measurements. All chemical procedures were carried out under clean laboratory conditions in the Isotope Cosmochemistry and Geochronology Laboratory (ICGL) at ASU. To ensure low procedural blanks, all reagents used were obtained or prepared as follows. Nitric acid (HNO_3_) was twice distilled from trace metal grade acid using the sub-boiling Savillex DST-1000 Acid Purification System. Hydrochloric acid (HCl) was distilled once from trace metal grade acid using the Savillex DST-1000. Water for acid dilutions and chromatography was purified from reverse osmosis (RO) water using a Millipore Super-Q® water purification system (resistivity ~ 18 MΩ.cm) followed by a Milli-Q® Element water purification system with a 0.1 µm final filter (resistivity > 18.2 MΩ.cm).

Iron purification was conducted via ion exchange column chromatography in the ICGL at ASU following procedures similar to those described previously^61^. Iron was purified using a Bio-Rad column packed with 1 mL of pre-cleaned anion exchange Bio-Rad AG1-X8 200-400 mesh resin. The resin was further cleaned using 0.5 N HCl followed by pre-conditioning with 6 N HCl. Aliquots of dissolved sample solutions (having a maximum of 100 μg of Fe per sample) were loaded onto the column in a 6 N HCl medium. Cations such as Na, Mg, Al, Ca, Ti, V, Cr, and Ni were eluted using 6 N HCl and then Fe was eluted with 0.5 N HCl. The Fe column yield was typically better than 98.5%. The average total procedural blank for all chemical processing including sample digestion and Fe purification was ~ 10 ng of Fe. Iron isotopes were measured with the Thermo Finnigan Neptune multi-collector inductively coupled plasma mass spectrometer (MC-ICPMS) at ASU using a jet sample cone and an H-skimmer cone in medium-resolution mode (i.e., with a mass resolving power ≥ 8500). The purified Fe samples and standards were analyzed at concentrations of 500 ppb doped with 500 ppb NIST Cu to correct for instrumental mass bias. Samples were introduced through an ESI Apex Q sample introduction system attached to a self-aspirating PFA nebulizer with an uptake rate of 100 µL/min. An uptake time of 90 s and a wash time between consecutive sample and standard measurements of 120 s were used. Typical sensitivity achieved for ^56^Fe signal was ~ 70 V/ppm Fe using a 10^11^Ω resistor. Data were collected by switching between two different cup configurations wherein ^53^Cr, ^54^Fe, ^56^Fe, ^57^Fe, ^58^Fe, and ^60^Ni isotopes were measured in parallel during the first integration of 8 s using the L4, L2, C, H1, H2, and H4 Faraday cups respectively. Subsequently, ^63^Cu and ^65^Cu were measured during the second integration of 4 s using the L1 and C Faraday cups respectively. Each run for a sample, standard or blank comprised 20 cycles. Measurements of ^53^Cr and ^60^Ni were used to correct for potential isobaric interferences of ^54^Cr and ^58^Ni on ^54^Fe and ^58^Fe, respectively. All data reduction was performed offline using the Iolite software. We corrected for instrumental mass fractionation using the exponential mass fractionation law such that the corrected isotope ratio (denoted by an asterisk) is given by:

3 $$\left( {\frac{{^{{\text{x}}} {\text{Fe}}}}{{^{{{54}}} {\text{Fe}}}}} \right)^{*} = { }\left( {\frac{{^{{\text{x}}} {\text{Fe}}}}{{^{{{54}}} {\text{Fe}}}}} \right)_{{{\text{measured}}}} \times \left( {\frac{{{\text{M}}_{{\text{x}}} }}{{{\text{M}}_{54} }}} \right)^{\upbeta }$$

where ^x^Fe is ^56^Fe or ^57^Fe, M_x_ and M_54_ are the atomic masses of ^x^Fe and ^54^Fe respectively and fractionation factor $\upbeta$ is defined by

4 $$\upbeta = \frac{{{\text{ln }}\left[ {\frac{{\left( {\frac{{^{{{63}}} {\text{Cu}}}}{{^{{{65}}} {\text{Cu}}}}} \right)_{{{\text{true}}}} }}{{\left( {\frac{{^{{{63}}} {\text{Cu}}}}{{^{{{65}}} {\text{Cu}}}}} \right)_{{{\text{measured}}}} }}} \right]}}{{{\text{ln}}\left( {\frac{{{\text{M}}_{63} }}{{{\text{M}}_{65} }}} \right)}}$$

where (^63^Cu/^65^Cu)_true_ is the true isotopic abundance ratio of ^63^Cu and ^65^Cu, and M_63_, M_65_ are the atomic masses of ^63^Cu and ^65^Cu respectively. Iron isotope compositions are reported using the $\delta$ notation, i.e., parts per thousand deviation (‰) relative to the bracketing standard IRMM-524A according to:

5 $$\delta^{x} {\text{Fe}}_{{{\text{sample}}}} ( {{\textperthousand}} ) = \left[ {\frac{{\left( {\frac{{^{{\text{x}}} {\text{Fe}}}}{{^{{{54}}} {\text{Fe}}}}} \right)^{*}_{{{\text{sample}}}} }}{{\left( {\frac{{^{{\text{x}}} {\text{Fe}}}}{{^{{{54}}} {\text{Fe}}}}} \right)^{*}_{{{\text{IRMM}} - 524{\text{A}}}} }} - 1} \right] \times 1000$$

To assess the accuracy and precision of our methodology for Fe isotope analyses (including chemical separation and mass spectrometry), we analyzed purified Fe from BCR-2, BIR, BHVO-1, and TAG-sulfide terrestrial rock standards as well as from a homogenized bulk sample of the Allende CV3 chondrite along with the aubrite metal samples during each MC-ICPMS analytical session. The Fe isotope composition reported for each sample is the average of six repeat runs and is reported with the internal 2SE uncertainty of these measurements or the long-term reproducibility, whichever is larger. The long-term reproducibility of our analyses based on repeated measurements of the IRMM-524A bracketing standard over the course of this study is 0.010‰ for δ^56^Fe and 0.015‰ for δ^57^Fe (Supplementary Fig. S2).

### Calculation of temperature of metal-silicate equilibration

The following relationship^34^ was used to calculate the metal-silicate equilibration temperature:

6 $${\text{log}}\left( {\frac{{x_{{{\text{Si}}}}^{metal} }}{{x_{{{\text{SiO}}_{2} }}^{silicate} }}} \right) = a + \frac{b}{{\text{T}}} + \frac{{c{\text{P}}}}{{\text{T}}} + d\frac{nbo}{t} - 2\log \left( {\frac{{x_{{{\text{FeO}}}}^{silicate} }}{{x_{{{\text{Fe}}}}^{metal} }}} \right){ } - \log \left( {\frac{{\gamma_{{{\text{Si}}}}^{metal} }}{{\left( {\gamma_{{{\text{Fe}}}}^{metal} } \right)^{2} }}} \right)$$

where $x_{i}^{phase}$ is the molar fraction of element *i* in the phase of interest and $\gamma_{i}^{phase}$ is the activity coefficient of component *‘i*’ in the phase of interest. The term *nbo/t* is the ratio of non-bridging oxygens to tetrahedrally coordinated cations in the silicate melt, P is the pressure in GPa, T is temperature in Kelvin, and *a*, *b*, *c*, and *d* are parameters obtained from the regression of experimental data^34^ (Supplementary Table S1). In their experiment^34^, these authors estimated *nbo/t* = 1.74 for a reduced enstatite chondrite-like starting composition, a value we also use for the similarly reduced aubrites. The terms $x_{{{\text{Si}}}}^{metal}$ and $x_{{{\text{Fe}}}}^{metal}$ for each metal grain have been calculated using their bulk elemental composition (Supplementary Table S2). The terms $x_{{{\text{SiO}}_{2} }}^{silicate}$ and $x_{{{\text{FeO}}}}^{silicate}$ for each aubrite have been calculated as the weighted average of SiO_2_ and FeO, respectively, in various silicate phases in that particular aubrite based on published data^62^ (Supplementary Table S2). We used $\gamma_{{{\text{Fe}}}}^{metal}$ = 0.8 as previously suggested^34^, for all our calculations. Uncertainties in T mostly represent uncertainties in the regression parameters^34^. The term $\gamma_{{{\text{Si}}}}^{metal}$ for each metal has been calculated using the following equation^63^:

7 $$\begin{aligned} \ln \gamma_{{{\text{Si}}}} & = \ln \gamma_{{{\text{Fe}}}} + \ln \gamma_{Si}^{0} - \varepsilon_{Si}^{Si} \ln \left( {1 - x_{Si} } \right) \\ & \quad - \mathop \sum \limits_{{j = 2\left( {j \ne {\text{Si}}} \right)}}^{N} \varepsilon_{Si}^{j}x_{j} \left( {1 + \frac{{\ln \left( {1 - x_{j} } \right)}}{{x_{j} }} - \frac{1}{{1 - x_{Si}}}} \right) \\ & \quad + \mathop \sum \limits_{{j = 2\left( {j \ne {\text{Si}}} \right)}}^{N} \varepsilon_{Si}^{j} x_{j}^{2} x_{Si} \left( {\frac{1}{{1 - x_{Si} }} + \frac{1}{{1 - x_{j} }} + \frac{{x_{Si} }}{{2\left( {1 - x_{Si} } \right)^{2} }} - 1} \right) \\ \end{aligned}$$

where $\gamma_{{{\text{Si}}}}$ is the activity coefficient of solute Si in the molten alloy made up of N constituents. For this study, based on our bulk elemental data for the metal grains (Table 1), we use three constituents (i.e., Ni, Co, and P) other than Si and Fe in the metal. The term $\gamma_{Si}^{0}$ is the Raoultian activity coefficient of solute Si at an infinite dilution in pure liquid Fe; $\varepsilon_{Si}^{j}$ is the first-order interaction parameter of solute *j* upon Si, and $x_{Si}$, $x_{j}$ are the mole fractions of Si and solute *j* in metal. Similar to the previous study^34^, we have extrapolated the terms $\gamma_{Si}^{0}$ and $\varepsilon_{Si}^{j}$ which were acquired at 1873 K to a temperature ‘T’, i.e., calculated $\ln \gamma_{Si}^{0} \left( T \right)$ and $\varepsilon_{Si}^{j} \left( T \right)$ according to the following equations:

8 $$\ln\gamma_{Si}^{0} \left( T \right) = \frac{{1873*\ln \gamma_{Si}^{0} }}{T}$$

9 $$\varepsilon_{Si}^{j} \left( T \right) = \frac{{1873*\varepsilon_{Si}^{j} }}{T}$$

Equations (8) and (9) and the values of $\gamma_{Si}^{0}$ and $\varepsilon_{Si}^{j}$ at 1873 K have been obtained from the Steelmaking Data Sourcebook^64^ and are presented in Supplementary Table S1.

### Calculation of oxygen fugacity

The following relationship was used to calculate the oxygen fugacity relative to the iron-wüstite (IW) buffer:

10 $$ \Delta \text IW = 2\log \left( {\frac{{x_{{{\text{FeO}}}}^{silicate} }}{{x_{{{\text{Fe}}}}^{metal} }}} \right) + 2\log \left( {\frac{{\gamma_{{{\text{FeO}}}}^{silicate} }}{{\gamma_{{{\text{Fe}}}}^{metal} }}} \right) $$

where $x_{i}^{phase}\;{\text{and}}\; \gamma_{i}^{phase}$ are the molar fraction and activity coefficient of component ‘*i’* in the phase of interest, respectively. The term $x_{{{\text{FeO}}}}^{silicate}$ for each aubrite was calculated as the weighted average of FeO in the silicate phases in that particular aubrite based on published data^62^ (Supplementary Table S2). We have used $\gamma_{{{\text{FeO}}}}^{silicate}$ = 3 ± 1 and $\gamma_{{{\text{Fe}}}}^{metal}$ = 0.8 after a previous study^34^. Uncertainties in *f*O_2_ primarily reflect the uncertainty in $\gamma_{{{\text{FeO}}}}^{silicate}$. A higher value for $\gamma_{{{\text{FeO}}}}^{silicate}$ (= 5.83) was used in a previous study^17^ to account for the high MgO content (~ 40 wt%) in aubrite silicates; using this value translates to *f*O_2_ in the range of –4.8 to –5.1 log units relative to the iron-wüstite buffer for aubrite metals studied here (compared to –5.4 to –5.7 estimated here; Table 1). Irrespective of the choice of $\gamma{_{{{\text{FeO}}}}^{silicate}}$ values, the calculated *f*O_2_ values vary little, suggesting that the primary control on Si content and δ^56^Fe of metal is temperature.

### Thermal modeling of the aubrite parent body

We have used the following equation^40^ for calculating the temperature profiles within the aubrite parent body at different times after its accretion:

11 $$T = T_{0} + \frac{{\kappa A_{0} }}{K\lambda }e^{ - \lambda t} \left[ {\frac{{Rsin\left( {r \sqrt {\frac{\lambda }{\kappa }} } \right)}}{{rsin\left( {R \sqrt {\frac{\lambda }{\kappa }} } \right)}} - 1} \right] + \frac{{2R^{3} A_{0} }}{{r\pi^{3} K}} \mathop \sum \limits_{n = 1}^{\infty } \frac{{ - 1^{n} }}{{n\left( {n^{2} - \frac{{\lambda R^{2} }}{{\kappa \pi^{2} }}} \right)}} sin\left( {\frac{n\pi r}{R}} \right)e^{{ - \frac{{\kappa n^{2} \pi^{2} t}}{{R^{2} }}}}$$

where $T_{0}$ is the ambient temperature in the disk at the time of accretion of the aubrite parent body; ${\text{K}}\;{\text{is}}\;{\text{thermal}}\;{\text{conductivity}};{ }\kappa$ is the thermal diffusivity defined as $\left( {K/\rho C_{P } } \right)$, where $\rho$ is density and $C_{P }$ is specific heat capacity; $A_{0}$ is the power output per unit volume at the time of planetesimal formation (see Supplementary Table S3 for details on the calculation of $A_{0}$); $\lambda$ is the decay constant of ^26^Al; t is the time elapsed since accretion; r is the distance from center; and R is the radius of the aubrite parent body. All parameters used in Eq. (11) above are given in Supplementary Table S3. Aubrites are suggested to have formed within ~ 1 AU of the Sun based on their reduced nature^65, 66^. As such, as a bounding case, we have used parameters appropriate for formation at 1 AU (Supplementary Table S3). We note that the accretion time of the aubrite parent body is not well-constrained. However, thermal modeling assuming ^26^Al as the primary heat source for core formation on the aubrite parent body suggests that it accreted at or before ~ 1.5 Myr following CAI formation^42^. The main uncertainties in the modeled accretion age are associated with the temperature of core formation and the Al concentration of the aubrite parent body^42^. Given the estimated upper limit on the accretion time of the aubrite parent body, we chose to perform our calculations assuming accretion times of 1.0, 1.3, and 1.5 Myr after CAI formation to illustrate the differences in the depth from surface and the time after CAI formation at which the aubrite parent body could have acquired the thermal gradient relevant to the range of metal-silicate equilibration temperatures recorded by the aubrite metals in this study. For each of these accretion times, the relevant ambient temperature (*T*_0_) at the time of accretion at 1 AU is assumed^67^ (Supplementary Table S3). In Fig. 3a–c, for an accretion time of 1.0, 1.3, and 1.5 Myr, respectively, after CAI formation, we have shown the calculated temperature (T in Kelvin) as a function of the distance from the center (r in km) of the aubrite parent body at two different times since CAI formation such that the temperature within the parent body reaches 1635 K (black solid line) (i.e., the highest metal-silicate equilibration temperature recorded in the aubrite metals studied here) and 1723 K (black dashed line) (i.e., the temperature beyond which complete silicate melting occurs such that the resulting convective motion would obliterate any thermal gradient).

We note that recent numerical modeling suggested a formation location of ~ 2 AU from the Sun for the aubrite parent body^67^. Using parameters relevant for formation at 2 AU (Supplementary Table S3) and accretion times of 1.0, 1.3, and 1.5 Myr after CAI formation, we have estimated the times at which the appropriate thermal gradient will be acquired by the aubrite parent body (Supplementary Fig. S4). As such, if the aubrite parent body formed at 2 AU, for the estimated latest accretion time of ~ 1.5 Myr after CAI formation, we show that the relevant thermal gradient will be attained by 2.5–2.6 Myr after CAI formation; this is well within the maximum time interval of ~ 4.3 Myr estimated for acquiring the appropriate thermal gradient if the aubrite parent body formed at 1 AU. We also note that our calculations described above are done assuming a canonical ^26^Al/^27^Al ratio^68–70^. However, if we assume a lower ^26^Al/^27^Al ratio for inner solar system solids^71^, the aubrite parent body would have to accrete within a few hundred thousand years after CAI formation to generate enough heat to reach a temperature up to ~ 1635 K in its interior. In such a case, a thermal gradient relevant to the range of metal-silicate equilibration temperatures recorded by the aubrite metals could be acquired within ~ 5 Myr after CAI formation.

## Supplementary Information

Supplementary Information.

## Data Availability

The datasets generated during and/or analyzed during the current study are included in the main text and supplementary information.
