# Publisher Correction: Correlated iron isotopes and silicon contents in aubrite metals reveal structure of their asteroidal parent body

**DOI:** 10.1038/s41598-021-03384-8

**Published:** 2021-12-07

**Authors:** Soumya Ray, Laurence A. J. Garvie, Vinai K. Rai, Meenakshi Wadhwa

**Affiliations:** 1 School of Earth and Space Exploration, Arizona State University, Tempe, 85287 USA; 2 Center for Meteorite Studies, Arizona State University, Tempe, 85287 USA

Correction to: *Scientific Reports* https://doi.org/10.1038/s41598-021-99160-9, published online 19 November 2021

The original version of this Article contained a typographical error in Equation 10, where the 'γ' variable was omitted and ‘FeO’ was incorrectly given as ‘Feo’.

$$\mathrm{\Delta I}W=2\mathrm{ log}\left(\frac{{x}_{\mathrm{Feo}}^{silicate}}{{x}_{\mathrm{Fe}}^{metal}}\right)+2\mathrm{ log}\left(\frac{{}_{\mathrm{Feo}}^{silicate}}{{}_{\mathrm{Fe}}^{metal}}\right)$$

now reads:

$$\mathrm{\Delta IW}=2\mathrm{ log}\left(\frac{{x}_{\mathrm{FeO}}^{silicate}}{{x}_{\mathrm{Fe}}^{metal}}\right)+2\mathrm{ log}\left(\frac{{\gamma }_{\mathrm{FeO}}^{silicate}}{{\gamma }_{\mathrm{Fe}}^{metal}}\right)$$

The original Article has been corrected.

