# Supplementary material for: Correlated iron isotopes and silicon contents in aubrite metals reveal structure of their asteroidal parent body
Source: Sci Rep. 2021 Nov 19;11:22552. doi: 10.1038/s41598-021-99160-9 (PMC8605012; doi:10.1038/s41598-021-99160-9)
Supplement: Supplementary file 1 — Supplementary Information. [file 41598_2021_99160_MOESM1_ESM.pdf]

Correlated iron isotopes and silicon contents in aubrite metals reveal structure of their asteroidal parent body

Soumya Ray[1*], Laurence A. J. Garvie[1,2], Vinai K. Rai[1], Meenakshi Wadhwa[1]
[1]School of Earth and Space Exploration, [2]Center for Meteorite Studies, Arizona State University Tempe-85287, USA.

Supplementary Table S1: Parameters used to calculate the metal-silicate equilibration temperature (T in Kelvin) of aubrite metals.

Parameters	Values
(*nbo/t*)*	1.74
a**	2.97
b**	–21800
c**	–11
d**	–0.24
γ_{Fe}^{metal}**	0.8
γ_{Si}^{0} †	0.0013 at 1873 K
ε_{Si}^{Si} †	0.103 at 1873 K
ε_{Si}^{Ni} †	0.005 at 1873 K
ε_{Si}^{P} †	0.09 at 1873 K

* For calculating T, *nbo/t* is the average value for all experiments with starting composition (SM-2) similar to E-chondrite. Values adapted from Table 2 of previous study[1].
** Values used[1].
† Values used[2].
The x_i^{phase} used for calculation of T for each metal is given in Supplementary Table S2.

Supplementary Table S2: Molar fraction (x_i^{phase}) of component 'i' in the phase of interest. The values for x_i^{metal} have been calculated from elemental composition given in Table 1. Values for $x_i^{silicate}$ have been calculated using published data[3]. For each meteorite, $x_i^{silicate}$ is the weighted average of the mole fractions of 'i' in various silicates present in that particular meteorite.

Samples	x_{Fe}^{metal}	x_{Si}^{metal}	x_{Ni}^{metal}	x_{Co}^{metal}	x_{P}^{metal}	$x_{SiO_2}^{silicate}$	$x_{FeO}^{silicate}$
NC2	0.908199	0.015875	0.066962	0.008254	0.000711	0.473208	0.000437
NC3	0.925137	0.007495	0.056820	0.009296	0.001252	0.473208	0.000437
NC4	0.932362	0.005701	0.053058	0.007097	0.001783	0.473208	0.000437
NC6	0.929859	0.006266	0.054816	0.007461	0.001598	0.473208	0.000437
NC7	0.904802	0.032682	0.054409	0.006279	0.001828	0.473208	0.000437
NC8	0.904239	0.027397	0.061400	0.005534	0.001430	0.473208	0.000437
NC9	0.892520	0.041172	0.058825	0.006426	0.001057	0.473208	0.000437
NC10	0.895092	0.034393	0.062727	0.006897	0.000891	0.473208	0.000437
NC11	0.911620	0.012726	0.067651	0.006380	0.001623	0.473208	0.000437
NC12	0.900899	0.014544	0.076937	0.005993	0.001626	0.473208	0.000437
ME1	0.900992	0.039138	0.051643	0.006985	0.001242	0.499335	0.000488
ME2	0.888494	0.038952	0.064678	0.006640	0.001236	0.499335	0.000488
ME3	0.890667	0.039088	0.062236	0.006599	0.001411	0.499335	0.000488
ME4	0.894279	0.040223	0.057184	0.006721	0.001593	0.499335	0.000488
BSP3	0.932745	0.001937	0.057272	0.006966	0.001080	0.525280	0.000459
SW1	0.919705	0.017365	0.049161	0.007331	0.006438	0.444562	0.000354
SW3	0.890702	0.017135	0.081975	0.005916	0.004271	0.444562	0.000354

Supplementary Table S3: Parameters used to calculate temperature (T in Kelvin) at different radial distances (r) from the center of the aubrite parent body using equation (11).

	Parameters	Value	Units	Reference
T_0	Ambient temperature	280* and 194** at 1.5 Myr† 305* and 208** at 1.3 Myr† 335* and 230** at 1.0 Myr†	K	[4]
K	Thermal conductivity	5 at 1 AU 5.3 at 2 AU	$Wm^{-1}K^{-1}$	[5,6]
ρ	Density of ECs	3550	kgm^{-3}	[7]
C_P	Specific heat capacity	800 at 1 AU 540 at 2 AU	$Jkg^{-1}K^{-1}$	[8]
κ	Thermal diffusivity	1.76×10^{-6} at 1 AU 2.76×10^{-6} at 2 AU	m^2s^{-1}	$K/\rho C_P$
A_0	Power output per unit volume	1.37×10^{-4} at 1.5 Myr† 1.67×10^{-4} at 1.3 Myr† 2.24×10^{-4} at 1.0 Myr †	Wm^{-3}	See below for details
λ	Decay constant of ^{26}Al	3.1170×10^{-14}	s^{-1}	*ln (*2) / $T_{1/2}$
$T_{1/2}$	Half-life of ^{26}Al	705000	yr	[9]
R	Radius of aubrite parent body	100	km	[10]

* T_0 in Kelvin at 1 AU from the Sun
** T_0 in Kelvin at 2 AU from the Sun
† Time of accretion after CAI formation

Calculation of the power output per unit volume at the time of planetesimal formation (A_0)

The energy per decay of ^{26}Al can be converted to the heat output (H_0) per unit mass in W/kg according to,

$$H_0 = \frac{N_A}{m_{26}} E_d e_v \lambda$$

where,

N_A (Avogadro's number) = 6.0221415×10^{23}
m_{26} (mass of 1 mole of ^{26}Al) = 25.987×10^{-3} kg
E_d (amount of energy released per decay) = 3.12 MeV[11]
$e_v = 1.60217646\times10^{-19}$ J/eV
λ (decay constant of ^{26}Al) = $3.1170\times10^{-14}\ s^{-1}$

Then, A_0 which is the power output per unit volume at the time of planetesimal formation in W/m^3 can be calculated according to,

$$A_0 = H_0 \rho x C e^{-\lambda t_{acc}}$$

where,
ρ (density) = 3550 kg/m^3 for enstatite chondrites[7].
x (weight fraction of Al in solid) = 0.00889; average value of enstatite chondrites[12].
C (canonical $^{26}Al/^{27}Al$ ratio) = 5.25×10^{-5} as shown in previous work[13].
t_{acc} (time of accretion of planetesimal relative to CAI formation); given that this is estimated to have an upper limit of ~1.5 Myr[14], we used values of 1.0, 1.3, and 1.5 Myr for illustrative purposes.

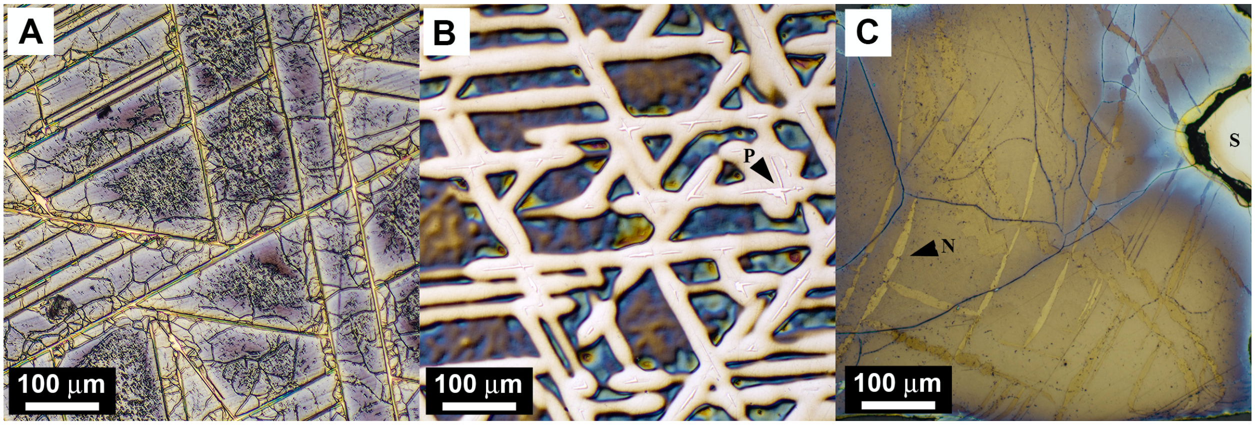

Supplementary Figure 1. Representative reflected light photographs of polished and stain-etched pieces of **A**) Norton County nodule NC8, **B**) Mount Egerton nodule ME1, and **C)** Bishopville nodule BSP3. The sodium bisulfite stain-etch colors the Ni-poor kamacite shades of tan-purple-blue, whereas high Ni-metal and the inclusions remain unstained and shiny. The Norton County nodule NC8 (**A**) clearly shows the linear inclusions of perryite that divide the surface into triangular and trapezoidal regions. A similar pattern is shown by the Mount Egerton ME1 nodule (**B**), though perryite only forms discontinuous linear grains (region P indicated by arrow) within Ni-rich linear metal (bright lath-like features). Bishopville is dominated by kamacite and lacks perryite. S – schreibersite, N – Neumann band.

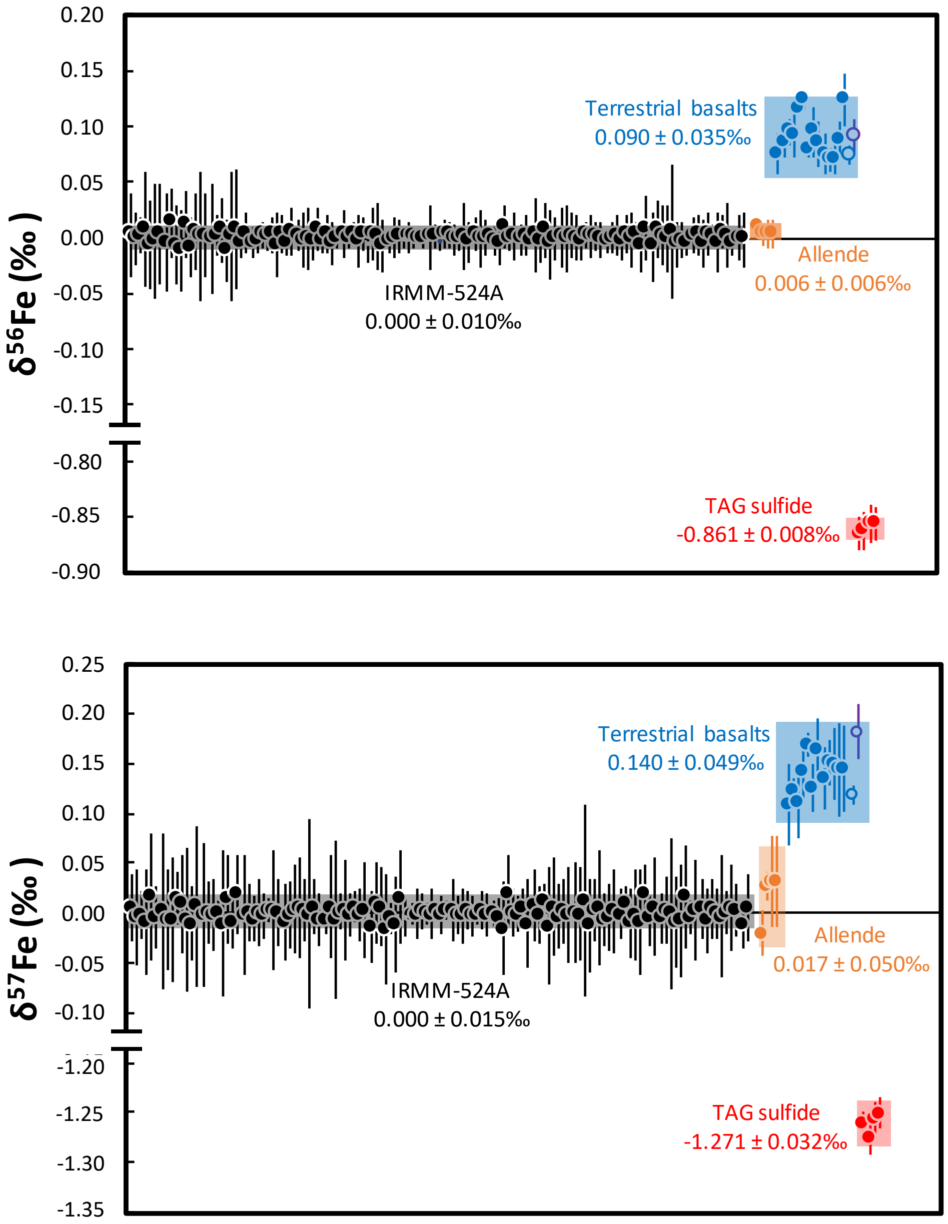

Supplementary Figure S2. Long-term external reproducibility of Fe isotope analyses in the Isotope Cosmochemistry and Geochronology Laboratory (ICGL) at ASU. Each data point represents an average of six repeat runs and is plotted with its 2SE uncertainty. Black circles show data for the IRMM-524A standard. Data for homogenized Allende powder is shown as orange circles; terrestrial basalt standards analyzed include BCR-2 (solid circles), BIR (blue open circle), and BHVO-1 (purple open circle); data for the Trans-Atlantic Geotraverse (TAG) sulfide are shown as the red circles. The shaded colored boxes represent the average (± 2SD) of all analyses for a given standard.

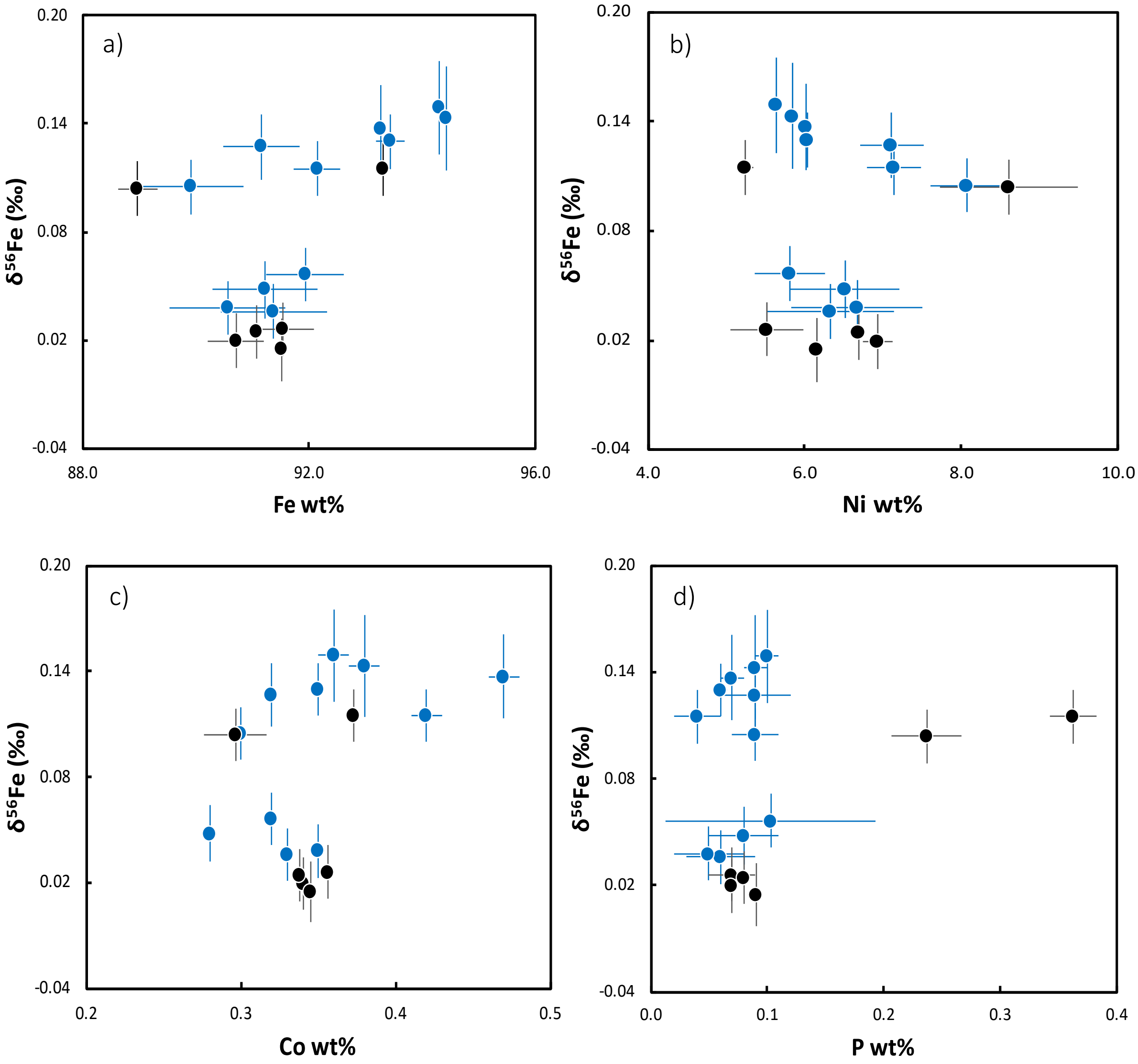

Supplementary Figure S3. δ^{56}Fe versus (a) Fe, (b) Ni, (c) Co, and (d) P content of the aubrite metals. Blue symbols are for metals from brecciated aubrites (Norton County and Bishopville) and black symbols are for metals from non-brecciated aubrites (Shallowater and Mount Egerton).

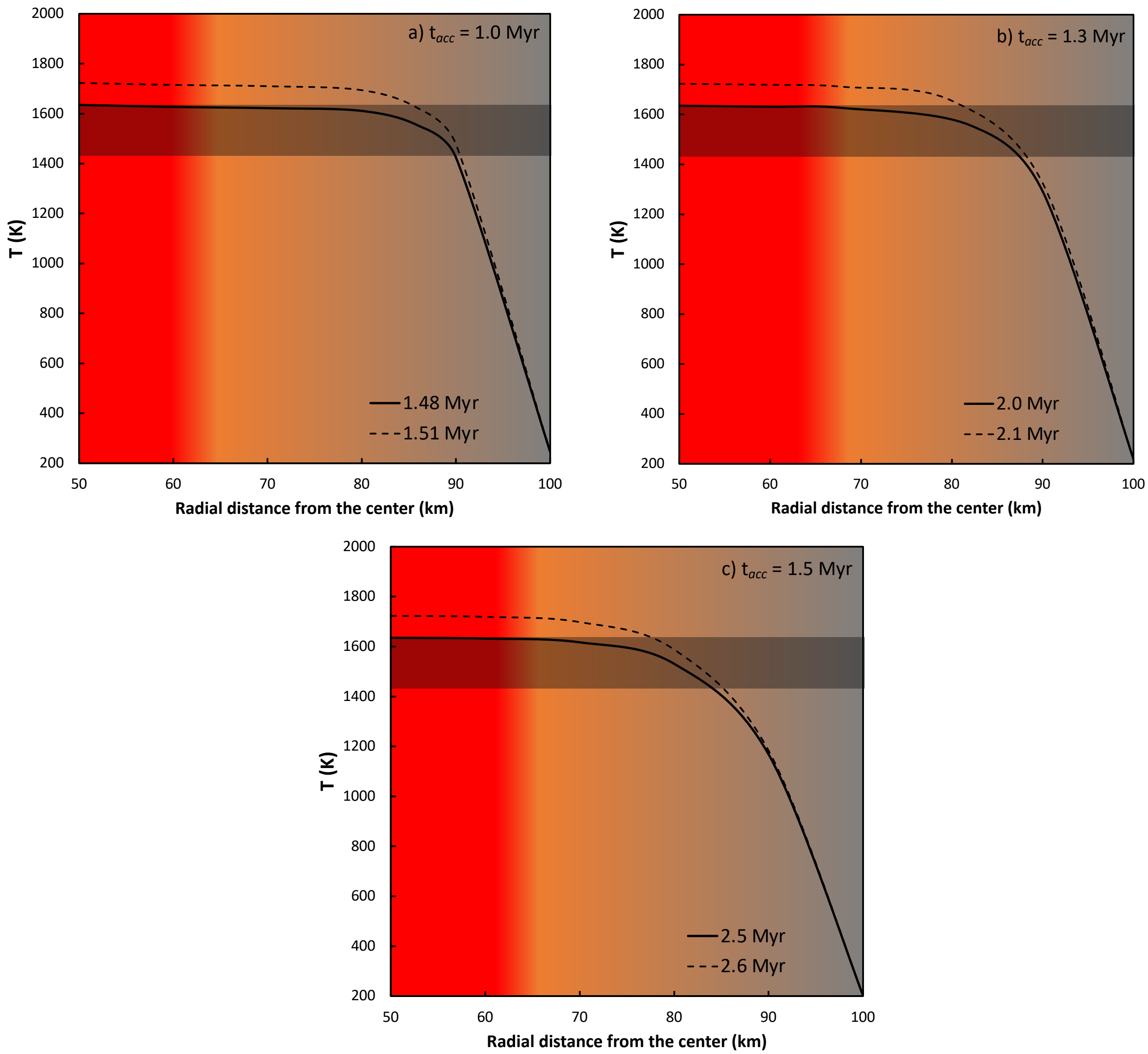

Supplementary Figure S4. The calculated temperature (T in Kelvin) versus radial distance (r in km) from the center of the 200 km diameter aubrite parent body at 2 AU from the Sun[4] at different times (shown by solid and dashed curves) assuming that the time of accretion (t_{acc}) was (a) 1.0 Myr; (b) 1.3 Myr; and (c) 1.5 Myr after CAI formation. The parameters used for these calculations are listed in Supplementary Table S3. Warmer colors represent higher temperatures with increasing depth from the surface. The black solid curve corresponds to the thermal profile with peak T of 1635 K (i.e., the highest estimated metal-silicate equilibration temperature recorded in the aubrite metals in this study). The black dashed curve represents the thermal gradient with peak T of 1723 K beyond which >50% silicate melting would lead to obliteration of the thermal gradient in the AuPB (see text for details). Horizontal gray bar illustrates the range of metal-silicate equilibration temperatures (1432−1635 K) estimated for the aubrite metals in this study. The thermal gradient within the AuPB inferred from the metal-silicate equilibration temperatures recorded by the aubrite metals in this study is attained by (a) 1.48–1.51 Myr (for t_{acc}= 1.0 Myr), (b) 2.0−2.1 Myr (for t_{acc}= 1.3 Myr), and (c) 2.5−2.6 Myr (for t_{acc}= 1.5 Myr) after CAI formation.

Supplementary References

1. Corgne, A., Keshav, S., Wood, B. J., McDonough, W. F. & Fei, Y. Metal-silicate partitioning and constraints on core composition and oxygen fugacity during Earth accretion. *Geochim. Cosmochim. Acta* **72**, 574–589 (2008).

2. The Japan Society for the Promotion of Science and The Nineteenth Committee on Steelmaking Part 2: Recommended values of activity and activity coefficients, and interaction parameters of elements in iron alloys. In *Steelmaking Data Sourcebook*. Gordon and Breach Science Publishers, New York. pp. 273–297 (1988).

3. Watters, T. R. & Prinz, M. Aubrites: Their origin and relationship to enstatite chondrites. *Proc. Lunar Planet. Sci Conf.* **X**, 1073-1093 (1979).

4. Desch, S. J., Kalyaan, A. & Alexander C. M. O. The Effect of Jupiter's Formation on the Distribution of Refractory Elements and Inclusions in Meteorites. *Astrophys. J. Suppl. Ser.* **238**, 11 (2018).

5. Opeil, C. P., Consolmagno, G. J. & Britt, D. T. The thermal conductivity of meteorites: New measurements and analysis. *Icarus* **208**, 449–454 (2010).

6. Opeil, C. P., Consolmagno, G. J., Safarik, D. J. & Britt, D. T. Stony meteorite thermal properties and their relationship with meteorite chemical and physical states. *Meteorit. Planet. Sci.* **47**, 319–329 (2012).

7. Macke, R. J., Consolmagno, G. J., Britt, D. T. & Hutson, M. L. Enstatite chondrite density, magnetic susceptibility, and porosity. *Meteorit. Planet. Sci.* **45**, 1513–1526 (2010).

8. Flynn, G. J., Consolmagno, G. J., Brown, P. & Macke, R. J. Physical properties of the stone meteorites: Implications for the properties of their parent bodies. *Chemie der Erde* **78**, 269–298 (2018).

9. Norris, T. L., Gancarz, A. J., Rokop, D. J. & Thomas, K. W. Half-life of ^{26}Al. *J. Geophys. Res. Solid Earth* **88**, B331–B333 (1983).

10. Wilson, L. & Keil, K. Consequences of explosive eruptions on small Solar System bodies: the case of the missing basalts on the aubrite parent body. *Earth Planet. Sci. Lett.* **104**, 505–512 (1991).

11. Castillo-Rogez, J., Johnson, T. V., Lee, M. H., Turner, N. J., Matson, D. L. & Lunine, J. ^{26}Al decay: Heat production and a revised age for Iapetus. *Icarus* **204**, 658–662 (2009).

12. Kong, P., Mori, T. & Ebihara, M. Compositional continuity of enstatite chondrites and implications for heterogeneous accretion of the enstatite chondrite parent body. *Geochim. Cosmochim. Acta* **61**, 4895–4914 (1997).

13. Kita, N. T. *et al.* ^{26}Al-^{26}Mg isotope systematics of the first solids in the early solar system. *Meteorit. Planet. Sci.* **48**, 1383–1400 (2013).

14. Sugiura, N. &Fujiya, W. Correlated accretion ages and $\varepsilon^{54}Cr$ of meteorite parent bodies and the evolution of the solar nebula. *Meteorit. Planet. Sci.* **49**, 772–787 (2014).
